# Clinician acceptance of complex clinical decision support systems for treatment allocation of patients with chronic low back pain

**DOI:** 10.1186/s12911-021-01502-0

**Published:** 2021-04-27

**Authors:** Stephanie Jansen-Kosterink, Lex van Velsen, Miriam Cabrita

**Affiliations:** 1 eHealth Group, Roessingh Research and Development, Roessinghsbleekweg 33b, 7522 AL Enschede, The Netherlands; 2 Faculty of Electrical Engineering, Mathematics and Computer Science, Telemedicine Group, University of Twente, Enschede, the Netherlands

**Keywords:** Clinical decision support system, Acceptance, Clinicians, Chronic low back pain

## Abstract

**Background:**

The uptake of complex clinical decision support systems (CDSS) in daily practice remains low, despite the proven potential to reduce medical errors and to improve the quality of care. To improve successful implementation of a complex CDSS this study aims to identify the factors that hinder, or alleviate the acceptance of, clinicians toward the use of a complex CDSS for treatment allocation of patients with chronic low back pain.

**Methods:**

We tested a research model in which the intention to use a CDSS by clinicians is influenced by the perceived usefulness; this usefulness, in turn is influenced by the perceived service benefits and perceived service risks. An online survey was created to test our research model and the data was analysed using Partial Least Squares Structural Equation Modelling. The study population consisted of clinicians. The online questionnaire started with demographic questions and continued with a video animation of the complex CDSS followed by the set of measurement items. The online questionnaire ended with two open questions enquiring the reasons to use and not use, a complex CDSS.

**Results:**

Ninety-eight participants (46% general practitioners, 25% primary care physical therapists, and 29% clinicians at a rehabilitation centre) fully completed the questionnaire. Fifty-two percent of the respondents were male. The average age was 48 years (SD ± 12.2). The causal model suggests that perceived usefulness is the main factor contributing to the intention to use a complex CDSS. Perceived service benefits and risks are both significant antecedents of perceived usefulness and perceived service risks are affected by the perceived threat to autonomy and trusting beliefs, particularly benevolence and competence.

**Conclusions:**

To improve the acceptance of complex CDSSs it is important to address the risks, but the main focus during the implementation phase should be on the expected improvements in patient outcomes and the overall gain for clinicians. Our results will help the development of complex CDSSs that fit more into the daily clinical practice of clinicians.

## Introduction

Clinical decision support systems (CDSSs) aid clinicians in decision-making during diagnosis, when referring to another healthcare professional, or to commence a treatment. CDSSs, defined as software that is designed to support clinical decision-making [1], have the potential to translate the most up to date knowledge and robust clinical evidence into practice [2]. Every CDSS is based on an input-process-output model. A classic or knowledge-based CDSS supports clinicians based on clinical guidelines. Nowadays, with the upsurge of big data, a CDSS can also generate feedback on quality indicators and treatment suggestions based on patient-specific information, guidelines, knowledge, stratification tools, and machine learning algorithms [2]. With the current, worldwide digitalization of healthcare and the increased use of electronic health records (EHR), more data is available to develop valid and reliable complex or non-knowledge based CDSSs [3]. Considering the current trend on patient empowerment [4], the use of CDSSs by clinicians increases the transparency of clinical decision-making and fosters the shared decision-making process between clinicians and patients [3]. Unfortunately, the impact of CDSSs use by clinicians on patient-related outcomes in daily clinical practice is inconclusive [5–7], as the fit between the CDSSs and clinical practice seems to be insufficient [8, 9]. And thus, despite the proven potential of CDSSs to reduce medical errors and to improve the quality of care, the uptake of these systems in clinical practice remains low [3, 10].

One of the frequently mentioned barriers for the uptake of complex CDSSs is the “black box” effect: clinicians are aware of the input and the output of complex CDSSs, but not of the decision-making algorithm within the CDSS [3, 11]. Next to this, the literature identifies various barriers to use CDSSs, such as ‘alert fatigue’ [7, 9, 12, 13], usability issues [7, 14], lack of integration of the CDSS with the workflow [8, 9], use of CDSS being perceived as too time-consuming [7, 8], and the use of CDSS perceived as distracting from the clinician-patient relationship [8, 14]. Besides all these barriers, clinicians recognize the usefulness of CDSSs as these systems do help them to consider a broader range of diagnoses and to ask more targeted questions [8]. Complex CDSSs embedded in the EHR are considered especially valuable, as these systems can make individualized recommendations regarding suitable interventions [2, 6]. Clinicians react more positively when they feel the CDSS is filling in gaps and stimulates further reasoning [15]. Additionally, younger clinicians seem to be more enthusiastic and less critical about CDSSs than their older colleagues [14]. Finally, CDSSs are more likely to succeed when both clinician and patient are involved in the decision-making process to empower the patient to be active in their own care [10].

Previous literature has mostly focused on the acceptance of classic CDSSs. For example, the GUIDES checklist suggests that stakeholders and users’ acceptance of a CDSS (sub-domain 1.3) is essential to increase the success of guideline-based CDSSs [16]. It is unknown whether or not clinicians will accept the more complex CDSSs that use stratification tools and machine learning (and thus greatly increase the black box effect). Within the H2020 Back-UP project, a complex CDSS is developed for personalized and faster evidence-based management of neck and low back pain [17]. In order to achieve the successful implementation of a complex CDSS, such as the Back-UP system, it is important to understand the attitude of clinicians towards such technology [11]; therefore, the aim of this study is to identify the factors (and their importance) that hinder or alleviate the acceptance of clinicians toward the use of a complex CDSS for treatment allocation of patients with chronic low back pain.

## Theoretical background

Theoretical approaches such as the technology acceptance model (TAM) [18] and the Unified Theory of Acceptance and Use of Technology (UTAUT) model [19] are the most common models used in research to investigate the acceptance of CDSS among clinicians [7, 9, 20]. In this study, we will focus on identifying factors that affect the acceptance (or intention to use) of a complex CDSS, besides the commonly investigated usefulness and ease of use. Models that predominantly rely on these factors have been criticized, as their explanatory power for predicting the acceptance of CDSS among clinicians can be questionable [21]. Clinicians tend to respond to information and communication technology (ICT) differently from other users [22, 23], while the most common models that explain technology acceptance are developed to explain the acceptance of software by general users. By going a level deeper than perceived usefulness for explaining intention to use, we aim to increase the explanatory power of our research. Therefore, next to the core TAM constructs we investigate four additional determinants known in the literature as facilitators and barriers for acceptance of classic CDSSs among clinicians: Perceived service benefits [24], Perceived service risks [24], Perceived threat to professional autonomy [23, 25, 26] and Trusting beliefs [27].

### Perceived service benefits and risks

In general, users tend to use, or not use, ICT to the extent they believe the ICT will help them to perform their job better [18]. For understanding the perceived usefulness of complex CDSS among clinicians better, our research model will include these factors: perceived service benefits and perceived service risks. Perceived service benefits and perceived service risks refer to the degree to which the new situation (in this case, the use of CDSS) is perceived as superior or inferior to the existing situation [24]. While the perceived service risks are known to relate to the intention to use ICT, the relation between perceived service benefits and the intention to use ICT can be deemed inconclusive [24].

### Perceived threat to professional autonomy

Professional autonomy is defined by Walter & Lopez, 2008 [23] as having control over the conditions, processes, procedures or content of work, which will not be possessed or evaluated by others. Professional autonomy is pinpointed as the central clinician characteristic that may be affected by an ICT, such as CDSSs [23, 25]. The literature suggests that the intention to use a CDSS depends whether or not this CDSS threatens the clinicians’ professional autonomy [23, 25, 26]. Next to this relationship, perceived threat to professional autonomy is an important antecedent to perceived usefulness in the context of clinicians acceptance of CDSS [23].

### Trusting beliefs

For the acceptance of technology, trust is an important construct; additionally, clinicians consider trust as an essential condition in the adoption of ICT [22]. In this study we see trust as a collection of beliefs. Trusting beliefs concerning a CDSS include the clinicians’ perceptions about the CDSS’ benevolence, integrity and competence [27]. According to McKnight et al., 2002 [27], benevolence belief is a clinicians’ perception that a CDSS acts in their interest; integrity belief is the perception that a CDSS adheres to a set of principles important for clinicians; and competence belief is a clinician’s perception that a CDSS has the ability, skills, and expertise to perform effectively.

## Research methodology and analysis

**Fig. 1:**
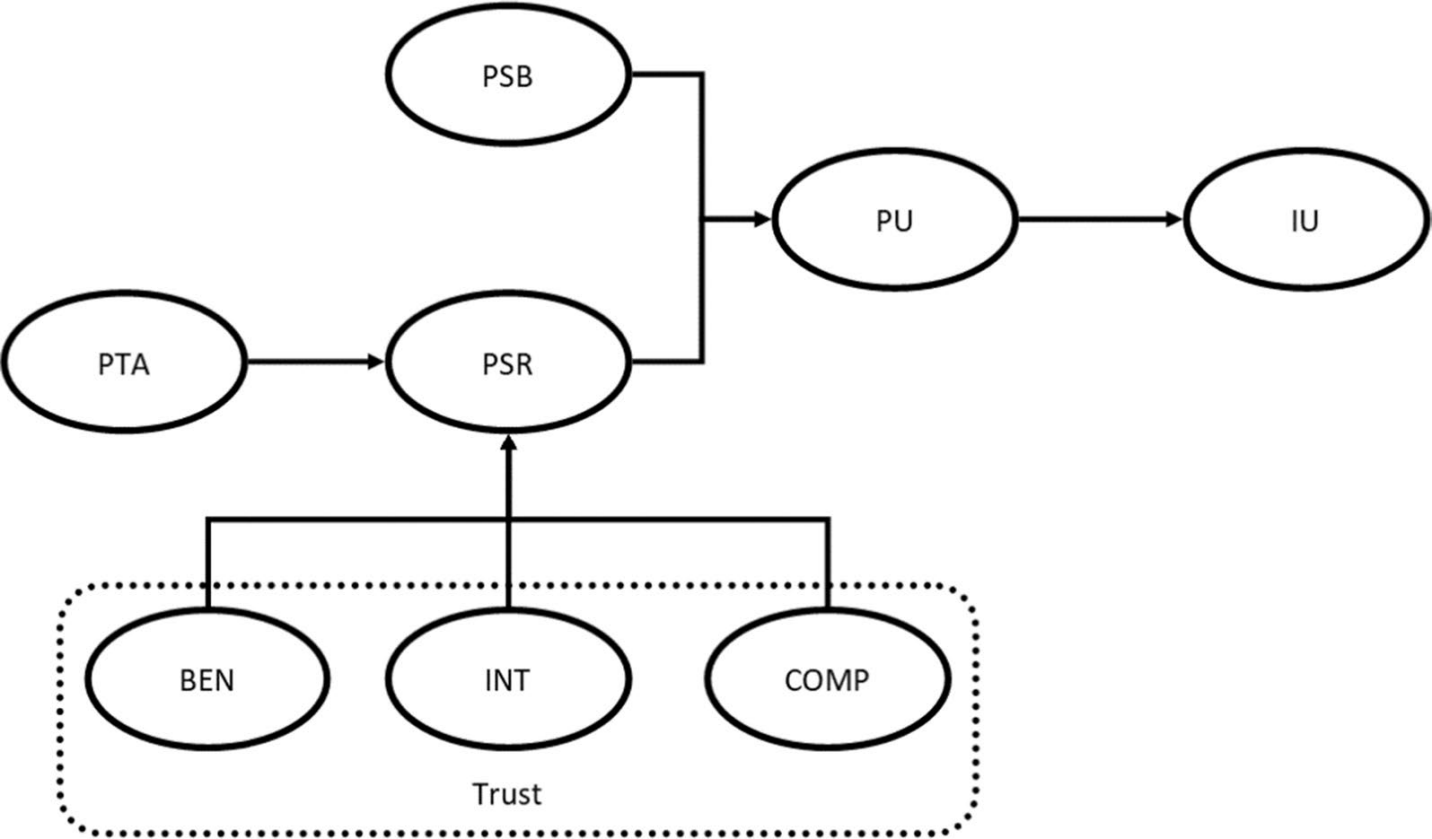
Research model

Figure 1 presents our research model, which is based on the literature previously discussed. The intention to use (IU) a CDSS by clinicians is predicted by perceived usefulness (PU), which, in turn, is shaded by the perceived service benefits (PSB) and risks (PSR). Risk, finally, is affected by the perceived threat to professional autonomy (PTA) and trusting beliefs (Trust). We adopted a survey method for data collection and tested our research model using Partial Least Squares Structural Equation Modelling (PLS-SEM).

### Measurement and data collection

Based on validated measurement scales we developed a survey. Table 1 provides an overview of the constructs and items that constitute the final version of the survey. For the construct Intention to use, three items were derived from van Velsen et al. [28]. The items to assess perceived usefulness were based on Peleg et al. [20]. Both the items of perceived service benefits as perceived service risk were derived from Hu et al. [24]. The six items to assess perceived threat to professional autonomy were based on Walter and Lopez [23]. For the constructs related to trusting beliefs (Benevolence, Integrity, and Competence) [27] no items related to (complex) CDSSs were available in the literature and are therefore composed by the authors, based on McKnight et al. [27]. Respondents were asked to indicate agreement with the items, using a five-point Likert scale of 1–5, ranging from 1 (“strongly disagree”) to 5 (“strongly agree”). The online questionnaire started with demographic and professional background-related questions: Gender, Age, Setting, Number of clinicians in practice, Number of years in practice, Experience with technology, and faith in general technology (adapted from McKnight et al. 2002 [27]). The online questionnaire ended with two open questions referring to the reasons to use and not use the CDSSs developed in the Back-UP project.

**Table 1:** Constructs and questionnaire items

Intention to Use (IU) [28]	IU1—If the Back-UP system would be available for me, I would definitely use it IU2—I would recommend the Back-UP system to others IU3—I hope that Back-UP becomes available to me
Perceived Usefulness (PU) [20]	PU1—Using the Back-UP system will help me to treat patients with back pain more efficiently PU2 -Using the Back-UP system will improve the quality of care that I will provide to patient with back pain PU3—Using the Back-UP system will ease the way in which I treat patient with back pain PU4—Using the Back-UP system will make my work more effective
Perceived Service Benefits (PSB) [24]	PSB1—Using the Back-UP system will improve the timeliness of patient care PSB2—Using the Back-UP system will reduce patient care and service costs PSB3—Using the Back-UP system will improve the service productivity of medical staff PSB4—Using the Back-UP system will reduce unnecessary patient transfers or admissions PSB5—Using the Back-UP system will improve overall effectiveness of patient care
Perceived Service Risks (PSR) [24]	PSR1—Using the Back-UP system will hinder physician – patient relationship PSR2—Using the Back-UP system will reduce patient care effectiveness PSR3—Using the Back-UP system will jeopardize patient privacy PSR4—Using the Back-UP system will bring psychological harm
Perceived Threat to professional Autonomy (PTA) [23]	PTA1—Using the Back-UP system will decrease my control over clinical decisions PTA2—Using the Back-UP system will decrease my professional discretion over patient care decisions PTA3—Using the Back-UP system will decrease my control over each step of the patient care process PTA4—Using the Back-UP system will increase monitoring of my diagnostic and therapeutic decisions by non-providers PTA5—Using the Back-UP system will decrease my control over the allocation of scarce resources PTA6—I find the Back-UP system advantageous for the medical profession as a whole
Benevolence (BEN) [27]	BEN1—I believe that the Back-UP system would act in my best interest BEN2—The Back-UP system is designed to help me
Integrity (INT) [27]	INT1—The Back-UP system will be honest in its advice INT2 -The advice the Back-UP system gives me is sincere
Competence (COMP) [27]	COMP1—The Back-UP system is competent and effective is providing advice COMP2—Overall, the Back-UP system is a capable and proficient advice provider

The study sample consisted of General Practitioners (GPs), primary care physical therapists, and clinicians working at a centre for rehabilitation. All respondents were working in the Netherlands and had full proficiency in the Dutch language. Respondents were eligible if they were 18 years of age or older and belonged to the target population. In our research model there is a maximum of three factors influencing another factor. Therefore, in order to achieve 80% statistical power at a significance level of 5%, a sample size of at least 59 participants would be necessary to detect a R^2^ of at least 0.25 [29]. The questionnaire was distributed online (via Qualtrics^XM^) between November 2019 and May 2020, using a snowball sampling via posts on social media (LinkedIn, Twitter, and Facebook) and personal connections. Due to the method of recruitment, a response rate could not be calculated. Following the Dutch legislation (Medical Research Involving Human Subjects Act (WMO)), the nature of this research did not require formal medical ethical approval. Before starting the online questionnaire, respondents were asked for their consent to use the data collected for research purposes.

### Animation CDSS

**Fig. 2:**
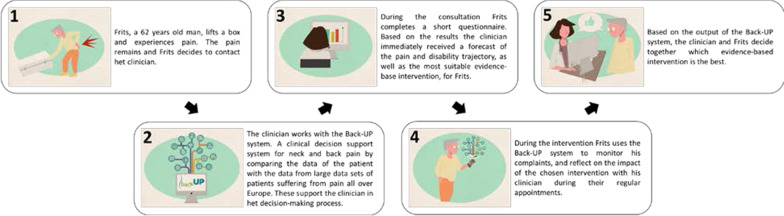
Visuals and text of the CDSS animation

The complex CDSS (developed within the H2020 Back-UP project to support personalized and faster evidence-based management of neck and low back pain) was presented to the respondents by a short animation without sound. The animation contained five frames and started with the introduction of Frits, a 62 years old male. Figure 2 provides an overview of the five frames’ visuals and texts. For every target population (i.e., GPs, primary care physical therapists, and clinicians working at a rehabilitation centre), the animation was slightly adopted to their clinical practice. Within the survey, this animation was in Dutch and presented to the respondents after the demographic questions and before the set of measurement items.

### Analysis method

We tested the relations presumed in the research model, via Partial Least Squares Structural Equation Modelling (PLS-SEM), using Smart PLS 3.0 [30] in two stages. In the first stage, we optimized the measurement model and assessed its quality. The first stage was divided into three steps. First, we determined the outer loadings of the indicators, as well as cross-loadings. Second, we assessed the reliability of the measurement scales by means of the composite reliability score, the Average Variance Extracted (AVE), and Cronbach’s alpha. Third, we checked for multicollinearity (via Variance Inflation Factor (VIF) scores) and determined whether the contribution of each individual indicator towards its construct was greater than 0 via outer weights and outer loadings in the formative model. In the second stage, we tested the causal model and determined the effect sizes of the significant causal relations by determining *f*^2^. To support the quantitative results, the responses on the two open questions were sorted and counted by the first author and discussed with the second author, taking a deductive approach. Disagreements were discussed until unanimous agreement was reached.

## Results

Of the 135 respondents who started the online questionnaire, 98 participants (73%) completed the full questionnaire. The two main reasons for participants not completing the questionnaire were lack of interest or time (18%), or being unable to watch the animation due to technical constraints their device (e.g. malfunction in specific internet browsers) (9%). Forty-six percent of the respondents were GPs, 25% worked as physical therapists in primary care and 29% worked as clinicians at a centre for rehabilitation. Fifty-two percent of the respondents were male and the average age was 48 years (SD ± 12.2). Most respondents worked together with 2 to 5 clinicians in their practice (38%) and worked as clinicians for 21 years or more (48%). Twenty-three percent of the respondents used a CDSS frequently in their clinical practice, forty-six percent used a CDSS occasionally, and thirty-one percent of the respondents had never used a CDSS in their clinical practice. The majority of the respondents (69%) showed neutral faith towards care technology. All demographic characteristics are presented in Table 2.

**Table 2:** Responders’ demographics (n = 98)

Gender	Male	52%
	Female	48%
Age in years		48.0 (SD ± 12.2)
Number of clinicians in practice	Only 1	9%
	2 to 5	38%
	6 to 10	15%
	11 to 20	7%
	21 or more	31%
Number of years in practice	Less than 1	1%
	1 to 5	10%
	6 to 10	17%
	11 to 20	24%
	21 or more	48%
Uses CDSSs during clinical practice	Frequently	23%
	Occasionally	46%
	Never	31%
Faith in care technology (Cronbach’s alpha = 0.8)		2.6 (SD ± 0.7)
	Positive	25%
	Neutral	69%
	Negative	6%

### Measurement model

The first step in determining the quality of our measurement model was to assess outer loadings (where items with an outer loading of > 0.7 were retained). All outer loadings exceeded this threshold, except for items of the perceived threat to professional autonomy scale and the perceived service risks scale. Item 1 to 5 of the perceived threat to professional autonomy scale had an outer loading < 0.4. When taking a look at the items that assessed this factor, we decided to first remove item 6 (*I find the Back-UP system advantageous for the medical profession as a whole*), as this item could also be considered to be an indicator of Perceived Usefulness. Removal of the item resulted in item 1 obtaining an acceptable outer loading. Next, we removed item 5 of the scale (*Using the Back-UP system will decrease my control over the allocation of scarce resources*) as it had a negative outer loading. After this removal, items 1 and 2 of the scale had an outer loading > 0.7. The item with the lowest outer loading (which was < 0.4), item 4, was then removed. Removal of this item resulted in a three-item scale, whereby items 1 and 2 had an outer loading > 0.7 and item 3 had an outer loading of > 0.5. Since the deletion of item 3 did not result in an increase of the scale’s Average Variance Extracted (AVE) and composite reliability, we retained this item.

Then, we focused on the perceived service risks scale. Two items had an outer loading > 0.7, while two items had an outer loading of > 0.5. Hence, the effect of removing one of the latter two items on AVE and composite reliability drove the decision to retain the items or not. First, we removed the item with the lowest outer loading score (item 3). Removal of the item slightly improved the construct’s composite reliability and greatly improved its AVE. The resulting items still contained one item with an outer loading of 0.6 (item 1). This item was removed. Again, a small effect on composite reliability and a large effect on AVE was the result. Therefore, we retained only items 2 and 4 for this construct.

For the remaining items, we assessed cross-loadings (see Table 3). All items load higher on the scale they are supposed to measure than on any other scale. This ensures the discriminant validity of the measurement model.

**Table 3:** Item cross loadings

	Latent variable							
	IU	PU	PSB	PSR	PTA	BEN	INT	COMP
IU1	**0.939**	0.662	0.547	− 0.581	0.222	0.436	0.401	0.436
IU2	**0.875**	0.479	0.401	− 0.420	0.137	0.375	0.379	0.368
IU3	**0.940**	0.673	0.521	− 0.482	0.116	0.455	0.406	0.372
PU1	0.598	**0.878**	0.630	− 0.561	0.283	0.421	0.381	0.555
PU2	0.638	**0.890**	0.549	− 0.540	0.264	0.378	0.377	0.458
PU3	0.534	**0.900**	0.678	− 0.616	0.377	0.434	0.334	0.422
PU4	0.616	**0.913**	0.757	− 0.570	0.344	0.432	0.378	0.433
PSB1	0.394	0.571	**0.869**	− 0.393	0.180	0.505	0.399	0.531
PSB2	0.414	0.634	**0.862**	− 0.435	0.204	0.490	0.363	0.496
PSB3	0.550	0.707	**0.853**	− 0.536	0.272	0.539	0.470	0.530
PSB4	0.469	0.602	**0.869**	− 0.409	0.160	0.464	0.398	0.473
PSB5	0.496	0.661	**0.897**	− 0.486	0.226	0.538	0.504	0.573
PSR2	− 0.598	− 0.576	− 0.534	**0.860**	− 0.259	− 0.531	− 0.515	− 0.555
PSR4	− 0.247	− 0.454	− 0.306	**0.762**	− 0.420	− 0.284	− 0.240	− 0.410
PTA1	− 0.165	− 0.342	− 0.194	0.378	**0.960**	− 0.099	− 0.073	− 0.281
PTA2	− 0.069	− 0.175	− 0.173	0.310	**0.746**	− 0.020	− 0.094	− 0.320
PTA3	0.037	− 0.013	− 0.070	0.171	**0.500**	0.113	0.058	− 0.077
BEN1	0.469	0.477	0.578	− 0.556	0.192	**0.932**	0.663	0.507
BEN2	0.352	0.351	0.467	− 0.354	− 0.015	**0.876**	0.596	0.445
INT1	0.397	0.378	0.446	− 0.444	0.070	0.662	**0.959**	0.632
INT2	0.430	0.410	0.501	− 0.481	0.135	0.683	**0.966**	0.600
COMP1	0.392	0.514	0.571	− 0.548	0.367	0.468	0.575	**0.931**
COMP2	0.407	0.459	0.548	− 0.573	0.274	0.519	0.618	**0.936**

Subsequently, we assessed the reliability of the different measurement scales by determining the composite reliability score, the AVE, and Cronbach’s alpha (Table 4). Thresholds for these scores are > 0.7 for composite reliability, > 0.5 for AVE, and > 0.7 for Cronbach’s alpha. All scores are acceptable to good, except for the Cronbach’s alpha value for perceived service risks, which is slightly below 0.7. However, since both AVE and the composite reliability score are acceptable for this construct, and since Cronbach’s alpha is a rather conservative reliability measurement for the case of a two-item construct, we will accept the reliability of this construct.

**Table 4:** Scale reliability

	Composite reliability	AVE	Cronbach’s alpha
Intention to Use	0.942	0.844	0.908
Perceived Usefulness	0.942	0.802	0.918
Perceived Service Benefits	0.940	0.757	0.757
Perceived Service Risks	0.795	0.661	0.661
Perceived Threat to professional Autonomy	0.793	0.576	0.799
Benevolence	0.900	0.818	0.781
Integrity	0.962	0.926	0.921
Competence	0.931	0.872	0.781

Next, we verified that there was no multicollinearity by determining the outer and inner Variance Inflation Factors (VIF) values. At this stage, we switched from reflective to formative model development. These values were all below the threshold of 5.00 (with a maximum value of 3.893 for outer VIF and a maximum value of 2.378 for inner VIF).

To end our assessment of the measurement model, we assessed the significance and relevance of the individual indicators, with respect to their latent variable. We used a bootstrapping procedure, using 5.000 samples to determine whether the contribution of each item towards its factor is significantly greater than 0. Eight outer weights were significant (p < 0.05) and therefore retained in the model. For the remaining 15 items, we looked at the outer loading. For all items, except perceived threat to autonomy items 2 and 3, the outer loading was > 0.5. For those two items, we looked at the significance of the outer loadings, but both were insignificant (p > 0.05). Therefore, they were removed from the model.

### Appreciation of factors

**Fig. 3:**
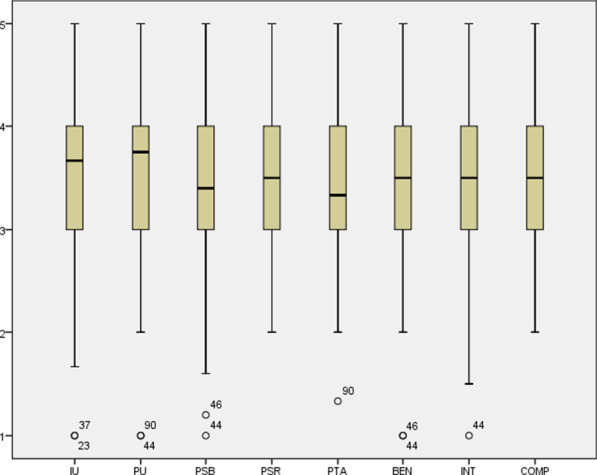
Boxplot of the factors of the measurement model

A boxplot (Fig. 3) presents the median score, quartiles and complete range of the factors of the measurement model. The average scores of these factors were 3.51 (SD 0.81) for Intention to use, 3.47 (SD 0.70) for Perceived Usefulness, 3.43 (SD 0.72) for Perceived Service Benefits, 3.47 (SD 0.66) for Perceived Service Risks, 3.35 (SD 0.74) for Perceived Threat to Autonomy, 3.53 (SD 0.66) for Benevolence, 3.53 (SD 0.69) for Integrity and 3.42 (SD 0.64) for Competence.

### Causal model

**Fig. 4:**
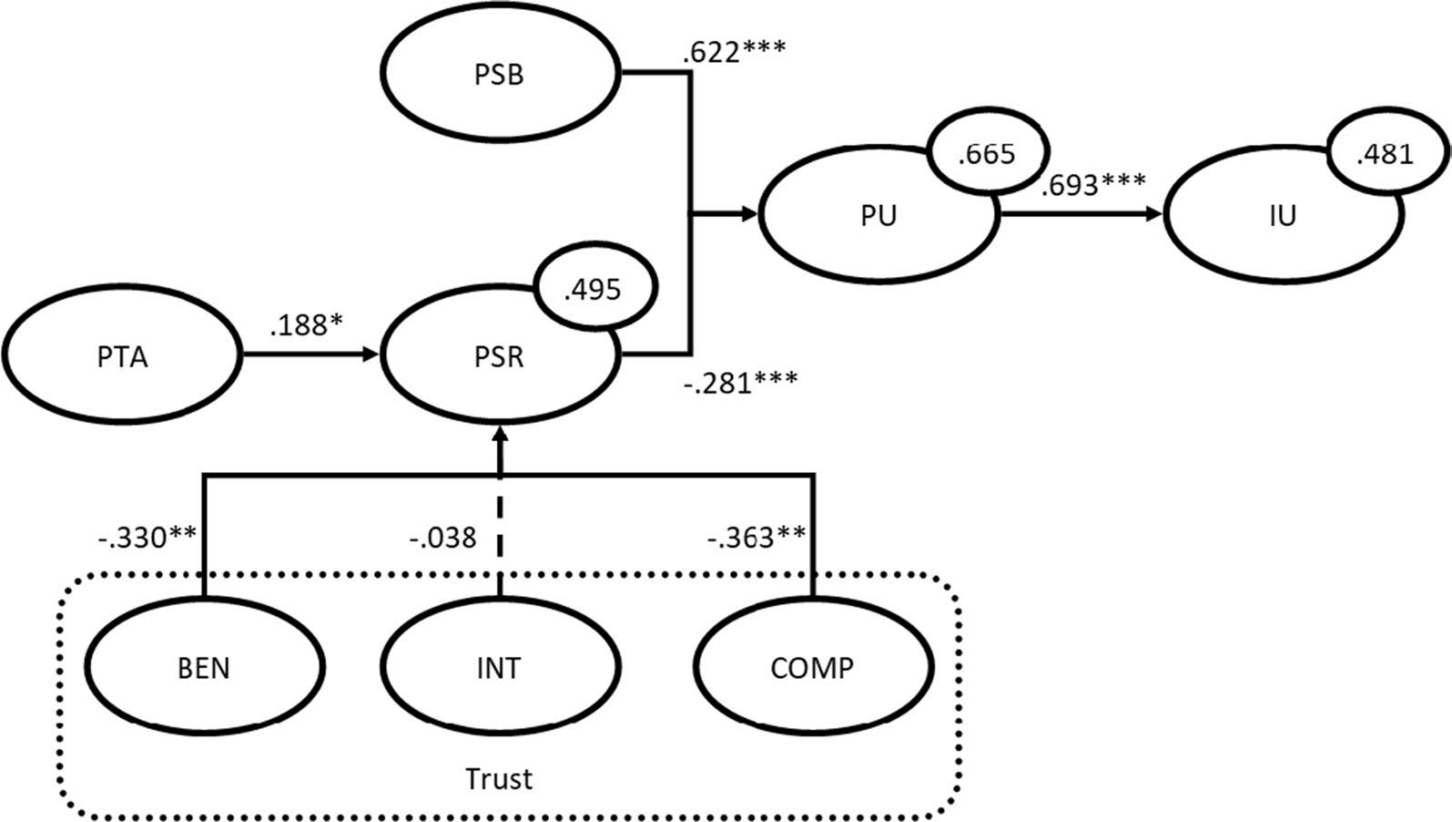
Causal model. *p < 0.0; **p < 0.01; ***p < 0.001

We assessed the causal model via a bootstrapping procedure with 5.000 bootstraps. The results can be found in Fig. 4.

Then we determined the effect size (*f*^2^) of the significant relations in the model [31]. These scores are as follows:

- Perceived Usefulness → Intention to Use: *f*^2^ = 0.926 (large effect size)
- Perceived Service Benefits → Perceived Usefulness: *f*^2^ = 0.778 (large effect size)
- Perceived Service Risks → Perceived usefulness: *f*^2^ = 0.159 (medium effect size)
- Perceived Threat to Professional Autonomy → Perceived Service Risks: *f*^2^ = 0.063 (small effect size)
- Benevolence → Perceived Service Risks: *f*^2^ = 0.115 (small effect size)
- Competence → Perceived Service Risks: *f*^2^ = 0.142 (small effect size)

### An overview of reasons to use and not use a complex CDSS

In line with the results of the quantitative study, the reason that was most often mentioned by respondents (n = 20) to use a complex CDSS when available was to improve the care for their patients, especially the assessment (e.g., *“Better streamlining of the right care.”, “Better treatment results for the client.*” and *“This can have a positive effect within the treatment of the patient.”*)*.* Second (n = 19), participants pointed out a curiosity to test and use the CDSS and see for themselves what the value of the system is (e.g., *“Curiosity, I would like to experience whether such a system can contribute to the treatment.”, “I am curious how this system works in practice” and “I would like to try the system to know the effect on patients and clinicians.”*). As the third most mentioned reason (n = 18), respondents expected an increase in efficiency as a result of the reduction of workload and time (e.g., “*Workload reduction, as the CDSS could also be used by the practice nurse*”,*”Time saving” and “Patients are informed by the system; this could make further consultation unnecessary”*). The use of a complex CDSS could help them to reorganize work. For instance, the supporting staff could ask the patient to complete the stratification questionnaire before a consult. As the fourth reason (n = 17), support during the decision making was mentioned (e.g., *“The CDSS can support and sharpen me in my own diagnostic thinking”, “The CDSS can help the healthcare professionals / team to make decisions about whether or not to follow treatment.” and “It seems useful to me to use [the system] in decision-making.”*). Patient empowerment is the fifth most often mentioned reason (n = 14) to use a complex CDSS (e.g., “*A nice way to see together with the patient whether the decision is wise*”, “*Clear policy information towards the patient. The patient has a clear picture of the possibilities and the patient can monitor himself.*” *and “The CDSS helps to provide the patient with general knowledge and insights regarding CLBP”)*. Next to these reasons, clinicians would use the tool to work consistently with evidence-based medicine (n = 8), and since they perceived the technology as friendly to use (n = 3).

As barriers to use the complex CDSS when available, respondents mentioned being worried about their own clinical practice and autonomy; they are reluctant to use a CDSS when it interferes too much with clinical practice (n = 18) (e.g., “*When the CDSS becomes leading and the clinical view of the practitioner is subordinated* “, “*When my role as a care provider is undermined or becomes more complicated*.”, and “*I would like to keep my own clinical reasoning without a CDSS.*”). Also, a large number of respondents do not want to use a CDSS when it comes at an increase in time and costs (n = 18) (e.g., “*Because it will take extra time that would be deducted from the time I have for my patient.*”, and “*Using the CDSS will cost more time in the beginning and learning to use the CDSS will cost time as well*”). The fear that the CDSS does not work correctly (n = 17) is also a reason not to use the CDSS (e.g., “*Too complicated to use for clinician and patient*”, *“Too cumbersome for the patient.” and “The tool is not easily accessible”.*) As the final reasons for not wanting to use the CDSS were a too generic approach (n = 15) (e.g., “C*ookbook medicine*”, “*No eye for specific patient characteristics*” and *“Insufficiently taking specific patient characteristics into account.”*), a lack of effectiveness and added value (n = 11) (e.g., “*The quality of the CDSS appears to be insufficient and not convincing of added value for the doctor and patient*.”, *“I think many years will come before the quality is so good that we can really rely on it.” and “If the CDSS has not been validated”),* hampering personal contact with the patient (n = 8) (e.g., “*Patients need attention and actual face-to-face contact*”, *“Less personal contact.” and “impersonal.”*), privacy and data security concerns (n = 8) (e.g., “*The privacy of the patient is not guaranteed.*”, *“Data security” and “What I think is important is that privacy must be properly guaranteed before it is put into use.”*), a capitalizing on healthcare (n = 4), lack of trust (n = 3), and if the use of CDSS is imposed by external parties, such as healthcare insurance companies (n = 3).

## Discussion

The aim of this study was to identify the factors, and their importance, that hinder or drive the acceptance of complex CDSS for treatment allocation of patients with chronic low back pain among clinicians. The causal model resulting from the data collected with an online survey suggests that perceived usefulness is the main factor contributing to the intention to use a complex CDSS, such as the Back-UP system. The relationship between perceived usefulness and the intention to use a classic CDSS has been demonstrated in various studies [9, 25, 26]. Our results add to this body of literature by suggesting that the intention to use complex CDSSs is also strongly influenced by the extent clinicians believe these systems will help them to perform their job better. Next to this, perceived service benefits and risks are both significant antecedents of perceived usefulness. Perceived service benefits positively affect perceived usefulness; while, an increase in perceived service risks negatively influences perceived usefulness. Similar results have been found in literature when investigating the acceptance of eHealth by Esmaeilzadeh et al., [24]. In our study, perceived service risks consisted of the perceived threat to autonomy and trusting beliefs, particularly benevolence and competence. In various studies perceived threat to professional autonomy is hypothesized and indicated as an important antecedent to perceived usefulness of CDSSs [23, 26] and/or the intention to use [23, 25]. In our final model, the perceived threat to professional autonomy also affects perceived service risk. However, this influence is relatively small when compared to the influence of benevolence and competence. The relatively weak influence of the perceived threat to professional autonomy to perceived service risk found in our study and that is not wholly consistent with other research [23, 25, 26] can be explained by the way the CDSS was presented to clinicians. In our study, a specific complex CDSS was presented to clinicians by means of a video animation, while in earlier studies a generic type of classic CDSS was presented based on only a definition. As such, we think that our model is more reliable.

The qualitative data analysed is in line with the quantitative assessment of our research model. The three main reasons for using a complex CDSS, we found, are to improve the care for their patient, out of curiosity toward the potential of a CDSS, and because of an expected increase in efficiency of care provision. Similar explanations for intention to use are mentioned in the literature [11]. In a study by Zheng et al., [32] clinicians were more favorable towards a system that provides clinical reminders for chronic disease and preventive care when the system improves performance, leading to better care and higher efficiency. The three main factors hindering the intention to use a CDSS, we found, are a too high interference with clinical practice, high costs and time expected of using the CDSS, and potential malfunctioning of the system. Interference with clinical practice or workflow is mentioned in an earlier study as a reason for not willing to use a classic CDSS [33]. The second main reason for not willing the use a complex CDSS is also in line with an earlier study [34] focussing on an internet-based system that interactively presents clinical guidelines at certain points of care. The main unfavourable response to this classic CDSS was that benefits are lost because it takes so long to use. In line with our results, poor usability is also mentioned in literature as a reason for not wanting to use a CDSS [7, 14].

Like any study, this one has some limitations. Due to our recruitment method (snowball sampling via social media) our sample could have been affected by a selection bias. Our sample was mainly composed of very experienced clinicians, as almost 50% of the respondents had more than 21 years of experience. Therefore, our results are based on the views of a somewhat skewed sample of clinicians, which might reduce the generalizability of our findings. Next to this, we used an animation to introduce the complex CDSS to the participants, as we thought this was more insightful than a general description (which most other studies use to gauge the acceptance of these technologies). For the participants, it may have been difficult to answer our questions based on an animation. For a more reliable opinion on the acceptance of a CDSS, it would have been important for clinicians to use the technology in their daily clinical practice. Therefore, we stress the importance of a follow-up study focused on actual use and adoption once the Back-UP system is ready for implementation into a daily clinical practice. Nonetheless, we think that our explanation of a CDSS was an improvement over the standard explanation that is given in this type of studies. Finally, we selected validated measurement scales for the online questionnaire. However, when the quality of our measurement model was determined it seems that the quality of the questions related to perceived threat to professional autonomy and perceived service risks was mediocre, as items needed to be deleted.

## Conclusion

This study was the first step in understanding the acceptance of clinicians toward a complex CDSS. The main message of the user acceptance and system adaptation design (UASAD) model of Khairat el al. [11] is to involve end-users early in the design and throughout the development of CDSSs to maximize user acceptance. Therefore, our results will help the developers of the complex CDSSs to develop a technology that fits perfectly into the daily clinical practice of clinicians. Next to this, our study teaches us that it is important to highlight the perceived benefits of using a CDSS to improve its acceptance, rather than explaining how the technology evades the risks involved. In the end, we are only on the verge of a technological revolution in healthcare, where smart technology aids clinicians, all with the goal of improving patient care. We can only reap the benefits of these tools, when the human actors find a way to accept them and integrate them into their care organization and daily care. We hope that this study contributes to this development.

## Data Availability

The datasets generated during the current study are not publicly available but are available from the corresponding author on reasonable request.
